# Chlorogenic Acid Ameliorates Experimental Colitis by Promoting Growth of *Akkermansia* in Mice

**DOI:** 10.3390/nu9070677

**Published:** 2017-06-29

**Authors:** Zhan Zhang, Xinyue Wu, Shuyuan Cao, Meghan Cromie, Yonghua Shen, Yiming Feng, Hui Yang, Lei Li

**Affiliations:** 1 Department of Hygiene Analysis and Detection, School of Public Health, Nanjing Medical University, Nanjing 211166, Jiangsu, China; zhanzhang@njmu.edu.cn (Z.Z.); frank_newyue@163.com (X.W.); csyflora@163.com (S.C.); fym950911@163.com (Y.F.); 18351996510@163.com (H.Y.); 2 Department of Environmental Toxicology, The Institute of Environmental and Human Health, Texas Tech University, 1207 Gilbert Drive, Lubbock, TX 79416, USA; meghan.cromie@ttu.edu; 3 Department of Gastroenterology, The Affiliated Drum Tower Hospital of Nanjing University Medical School, Nanjing 210008, Jiangsu, China; Syh_19861229@163.com

**Keywords:** chlorogenic acid, colitis, inflammation, gut microbiota, *Akkermansia*

## Abstract

Chlorogenic acid (ChA)—one of the most abundant polyphenol compounds in the human diet—exerts anti-inflammatory activities. The aim of this study was to investigate the effect of ChA on gut microbiota in ulcerative colitis (UC). Colitis was induced by 2.5% dextran sulfate sodium (DSS) in C57BL/6 mice, which were on a control diet or diet with ChA (1 mM). The histopathological changes and inflammation were evaluated. Fecal samples were analyzed by 16S rRNA gene sequencing. ChA attenuated several effects of DSS-induced colitis, including weight loss, increased disease activity index, and improved mucosal damage. Moreover, ChA could significantly suppress the secretion of IFNγ, TNFα, and IL-6 and the colonic infiltration of F4/80^+^ macrophages, CD3^+^ T cells, and CD177^+^ neutrophils via inhibition of the active NF-κB signaling pathway. In addition, ChA decreased the proportion of *Firmicutes* and *Bacteroidetes*. ChA also enhanced a reduction in fecal microbiota diversity in DSS treated mice. Interestingly, ChA treatment markedly increased the proportion of the mucin-degrading bacterium *Akkermansia* in colitis mice. ChA acted as the intestine-modifying gut microbial community structure, resulting in a lower intestinal and systemic inflammation and also improving the course of the DSS-induced colitis, which is associated with a proportional increase in *Akkermansia*.

## 1. Introduction

Ulcerative colitis (UC) is one of the most common chronic inflammatory bowel diseases (IBD) of unknown etiology [1]. It is characterized by persistent progression or relapsing inflammation that mainly involve the colonic mucosa and submucosa. IBD is also associated with dysbiosis of the gut microbiota, such as a loss in bacterial diversity and shifts in the microbiota [2,3]. UC patients showed reductions in the amount of bacterial groups from the *Clostridium* cluster XIVa, and significantly higher levels of *Bacteroidetes* as compared with a healthy control [4]. The chemically-induced dextran sulfate sodium (DSS) colitis model has been shown to mimic human UC pathology with abnormal cytokine production and an intensive infiltration of neutrophils and macrophages into the colon epithelium [5,6]. The amount of *Lactobacillus* significantly decreases, while the amount of *Desulfovibrio* significantly increases in the DSS-treated mice [7]. A major reduction in *Bacteroidetes*/*Prevotella* and a corresponding increase in *Bacillaceae* were observed in the DSS-treated mice [8].

Chlorogenic acid (ChA)—a natural polyphenol product found in various plants—has anti-inflammatory and antiviral activities [9,10,11]. Coffee is a major source of chlorogenic acid that can be found in the diet with a daily intake of about 0.5 to 1 g in habitual coffee drinkers [12,13]. Epidemiological studies of IBD indicate that coffee consumption is a protective factor for UC in Asian-Pacific countries [14]. The anti-inflammatory effect of ChA on DSS-induced colitis symptoms, such as body weight loss, diarrhea, fecal blood, and shortening of colon were observed in C57BL/6 mice [15]. Since ChA was co-administered with DSS, ChA might diminish the impact of DSS on UC development in mice. However, the effects of ChA on cytokine production and cell infiltration are still unknown. In addition, microbial and immunological changes appear before the development of severe inflammation in the colons of mice treated with DSS [16]. In an in vitro mixed culture model of human intestinal microbiota, the fermentation of the ChA-stimulated proliferation of *Bifidobacteria* increased the production of short chain fatty acid (SCFA) when compared with controls [17]. Thus, it will be helpful to explore the effect of ChA on fecal microbiota in DSS-induced colitis to elucidate its anti-inflammatory activity.

The objective of the present study was to demonstrate the anti-inflammatory effect of ChA on DSS-induced acute colitis induced by DSS in C57BL/6 mice by macroscopic and histological methods. The levels of cytokine in serum, as well as colonic infiltration of immune cells, were evaluated. Moreover, the effect of ChA on gut microbiota of mice that were treated with DSS was also studied.

## 2. Materials and Methods

### 2.1. Animal Treatment

Female C57BL/6 mice (18–20, Shanghai SLAC Laboratory Animal Co., Ltd., Shanghai, China). The mice were maintained on a 12/12 h light/dark cycle at ambient temperature (22 °C) and 55% humidity. Five mice were placed in a plastic Macrolon cage with stainless steel covers and wood shaving, and had free access to standard mouse chow and tap water. The mice were allowed to acclimate for one week before the study began. The care and use of the animals were followed the animal welfare guidelines, and all the experimental protocols were approved by the Animal Care and Welfare Committee of Nanjing Medical University (FWA00001501).

### 2.2. Induction of Experimental Colitis and Chlorogenic Acid Treatment

As shown in Figure S1, animals were randomly divided into three groups (*n* = 10 for each group). Colitis was induced with dextran sulphate sodium (DSS, molecular weight of 36–50 kDa; MP Biomedicals Solon, OH, USA). Female mice were selected as they were less susceptible to DSS-induced disease and mortality than males [18]. The DSS group was given autoclaved water for the first seven days, and then 2.5% DSS for the last eight days. The DSS+ ChA group was given water containing 1 mM chlorogenic acid (ChA, purity ≥98.0%, Sigma, St. Louis, MO, USA) for 15 days—and the water also contained 2.5% DSS starting the eighth day. The control group was given autoclaved water for 15 days. The body weight was measured daily. The blood samples were collected by exsanguination via cardiac puncture on Day 15. They were allowed to clot at room temperature for two hours before centrifugation (3000× *g*, 4 °C, 10 min), and the serum was collected and stored at −80 °C for later use. At necropsy, the colon length was measured from the ileocecal junction to the anus. DSS-induced colitis was scored as the disease activity index (DAI) as described in our previous study [19]. Briefly, the DAI was the sum of weight loss (0, none; 1, 0–5%; 2, 5–10%; 3, 10–20%; and 4, >20%), stool consistency change (0, none; 2, loose stool; and 4, diarrhea), and bleeding (0, none; 1, trace; 2, mild hemoccult; 3, obvious hemoccult; and 4, gross bleeding), divided by three. The animals were scored for the DAI at the same time each day, blinded to the treatment.

### 2.3. Histology Assessment and Mucous Layer Analyses

Colon sections from distal (1–2 cm from the anal verge) segment were fixed in at 10% of the neutral buffered formalin, paraffin embedded, and stained with hematoxylin and eosin for examination by Pannoramic digital slide scanners (Pannoramic SCAN, 3DHISTECH Kft, Budapest, Hungary). The colon sections were stained with alcian blue, quantitative analyses for mucous production around epithelial limen were performed using ImageJ software according to a previous study [20].

### 2.4. Immunohistochemistry and Immunoblotting Assay

The immunohistochemistry assay was performed according to our previous study [21]. The expressions of CD 3, CD177, or F4/80 were determined the immunochemistry assay. Immune complexes were detected by enhanced chemiluminescence (ECL) using specific antibodies including p-P65, P65, and glyceraldehyde-3-phosphate dehydrogenase (GAPDH). Antibodies that were specific for CD3, CD177, F4/80, and GAPDH were obtained from Santa Cruz Biotechnology (Santa Cruz, CA, USA). Antibodies that were specific for p-P65, P65, and an ECL immunoblotting assay kit were purchased from Cell Signaling Technology (Danvers, MA, USA). For the densitometric analysis, the protein bands on the blots were measured using Image J software.

### 2.5. Multiplex Serum Cytokine Profiling

The serum levels of IFN-γ, TNF-α, IL-1β, IL-4, IL-6, and IL-12 were changed in the DSS treated mice in our previous study [19]. Thus, they were measured using MILLIPLEX™ micro-beads arrays (Millipore, Billerica, MA, USA) run on Luminex MAPIG instrument following the manufacturer’s recommended protocols. For evaluation of the results, Milliplex™ Analyst v5.1 (Vigenetech) was used. The median fluorescent intensity data were saved and analyzed using a five-parameter logistic or spline curve-fitting method for calculating cytokine concentrations in the samples. The coefficient of quality control variation of IFN-γ, TNF-α, IL-1β, IL-4, IL-6, and IL-12 were less than 2.17, 2.36, 4.71, 2.24, 4.18, and 5.92%, respectively.

### 2.6. Fecal Bacteria and Bioinformatics Analysis Using16S rRNA Gene High-Throughput Sequencing

To obtain enough parallel fecal samples in each group for a 16S rRNA gene high-throughput sequencing, 10 mice from each group were randomly divided into five cages on Day 14, the stools in each cage were collected on Day 15 before sacrifice. Bacterial DNA was extracted using TIANamp stool DNA kit (TIANGEN Biotech CO., Ltd., Beijing, China). Bacterial 16S rRNA at the V3 hypervariable region was amplified using a set of primers (338F: 5′-GTGCCAGCMGCCGCGGTAA-3′ and 806R: 5′-GGACTACHVGGGTWTCTAAT-3′). All polymerase chain reaction (PCR) recations contained 10 ng DNA template, TransStart FastPfu Polymerase (Transgen Biotech, Beijing, China) and forward primer and reverse primer at a final concentration of 200 nM. All reactions were carried out on a GeneAmp^®^ system 9700 (Applied Biosystems., Life Technologies, Warrington, UK) under the following cycling conditions: 95 °C for three min, followed by 27 cycles of 95 °C for 30 s, 55 °C for 30 s, 72 °C for 45 s, and a final extension step at 72 °C for 10 min.

Sequencing was performed by an Illumina MiSeq (PE300). Low quality sequences (<Q20), sequences that were shorter than 50 bp, and homopolymers that were longer than 10 bp and containing ambiguous base calls or incorrect primer sequences were removed. Paired-end sequences were merged to give an optimal alignment (overlap length ≥10 bp, mismatch proportion ≤20%). Sequences were clustered into operational taxonomic units (OTUs) using Mothur. The OTUs that reached 97% nucleotide similarity level were used for alpha diversity (Shannon and Simpson index), richness (ACE and Chao1), and rarefaction curve analyses using Mothur. There were 29,441 reads in each sample. The heatmap was generated on the basis of the relative abundance of OTUs using the R Project for Statistical Computing (version 2.15). Bray–Curtis dissimilarities were calculated from OTUs, following by the principal coordinate analysis (PCoA). Taxonomy was assigned using Ribosomal Database Project (RDP) database (http://rdp.cme.msu.edu/) with a 70% bootstrap score [22].

### 2.7. Akkermansia Quantification

To confirm the effects of ChA on *Akkermansia*, we used a group of mice that was treated with ChA for only seven days. *Akkermansia* was quantified with qPCR in accordance to previous study [23]. qPCR was done using 16S rRNA primers for *Akkermansia*: Forward CAGCACGTGAAGGTGGGGAC, reverse CCTTGCGGTTGGCTTCAGAT. Total 16S rRNA was also quantified and used to normalize *Akkermansia* using bacterial universal primers: Forward ACTCCTACGGGAGGCAGCAG and reverse ATTACCGCGGCTGCTGG. PCR products were analyzed by electrophoresis in a 2% agarose gel.

### 2.8. Statistical Analysis

The difference in body weight and DAI was analyzed using one-way analysis of variance (ANOVA) using SPSS 13.0 software (Chicago, IL, USA). A Mann–Whitney U test was used to assess the differences in cytokines and taxonomy of fecal microbiota. A *p*-value of less than 0.05 was considered significant.

## 3. Results

### 3.1. Dietary ChA Improves the Disease Activity Index (DAI) of DSS-Colitis Mice

**Figure 1:**
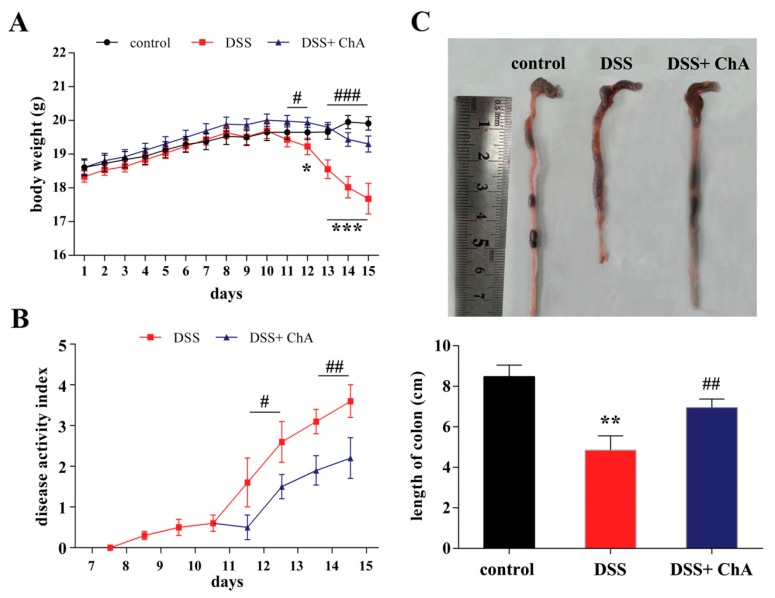
The effects of Chlorogenic acid (ChA) on the disease activity index (DAI) in dextran sulfate sodium (DSS) colitis mice. (**A**) Data for weight changes are expressed as the mean percentage change from the starting body weight; (**B**) Disease activity index was evaluated as the average of score of clinical parameters as body weight changes, rectal bleeding, and stool consistency or diarrhea; (**C**) Colon length of each group. The data are expressed as the mean ± SD from 10 mice in each group. * *p* < 0.05, ** *p* < 0.01, *** *p* < 0.001, compared with control group; ^#^ *p* < 0.05, ^##^ *p* < 0.01, ^###^ *p* < 0.001, compared with the DSS group according to one-way analysis of variance (ANOVA) statistical analysis.

Four days after colitis was induced by DSS treatment, all the mice showed apparent diarrhea and rectal bleeding. Significant loss of body weight was observed after the fifth day of DSS treatment, but ChA treatment could mend this loss in body weight (Figure 1A). The DAI in the DSS group steadily increased until the cessation of DSS treatment and the ChA supplementation attenuated diarrhea and rectal bleeding (Figure 1B). As an objective measure of the severity of inflammation, the colon length was measured. ChA treatment significantly improved the colon length versus the DSS group (Figure 1C). It suggested that ChA could significantly promote the recovery from the colitis.

### 3.2. The Effect of ChA on Histopathological Changes and the Infiltration of Inflammatory Cells in the Colon of the DSS-Colitis Mice

**Figure 2:**
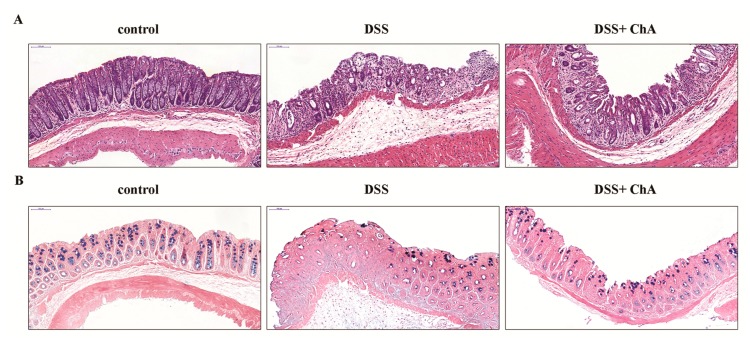
The effects of ChA on the histopathological characterization in DSS-colitis mice. (**A**) Representative HE-stained sections of the distal colonic tissues from the control, DSS, and DSS+ ChA group; (**B**) Representative alcian blue-stained sections of these different groups. The mucus is stained blue. Formalin fixed, paraffin-embedded 5 μm cross-sections were stained with respective primary antibody. Scale bar: 50 μm.

The histological evaluation of colonic tissue from healthy mice revealed a normal structure without histological changes. By contrast, mice that were treated with DSS exhibited serious injuries, with the loss of histological structure, and a strong epithelial disintegration, disruption of the epithelial barrier, a pronounced decrease in a number of crypts, and marked infiltration of granulocytes and mononuclear cells into the mucosa and submucosa (Figure 2A). ChA reduced the extent and the severity of the macroscopic and histological signs of colon injuries (Figure 2A). The depletion of mucin production was observed in DSS-induced colitis, and ChA supplementation could attenuate this effect (Figure 2B).

### 3.3. ChA Reduced Serum Cytokines in DSS-Colitis Mice

**Figure 3:**
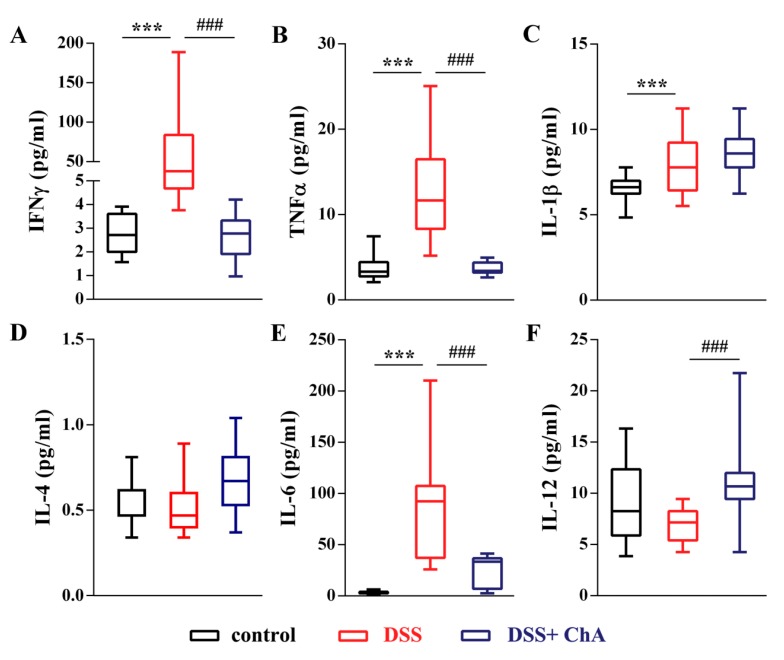
The effects of ChA on serum cytokines in DSS-colitis mice. A comparison of the serum concentration of (**A**) IFNγ; (**B**) TNF α; (**C**) IL-1β; (**D**) IL-4; (**E**) IL-6; and (**F**) IL-12 were performed by the Mann–Whitney U test. The boxplot represented the values of cytokines from minimum to maximum from 10 mice, *** *p* < 0.001 compared with control group; ^###^ *p* < 0.001, compared with the DSS group.

To determine the anti-inflammatory effect of ChA on DSS-induced colitis, the levels of six cytokines were measured in parallel following the induction of colitis (Figure 3). A substantial increase of IFNγ (Figure 3A), TNFα (Figure 3B), IL-1β (Figure 3C), and IL-6 (Figure 3E) was observed in DSS-treated mice. ChA treatment significantly reduced the serum level of IFNγ, TNFα, and IL-6. In addition, ChA significantly increased the level of IL-12 in DSS-induced colitis (Figure 3F).

### 3.4. ChA Attenuated DSS-Induced Colonic Infiltration of Inflammatory Cells

**Figure 4:**
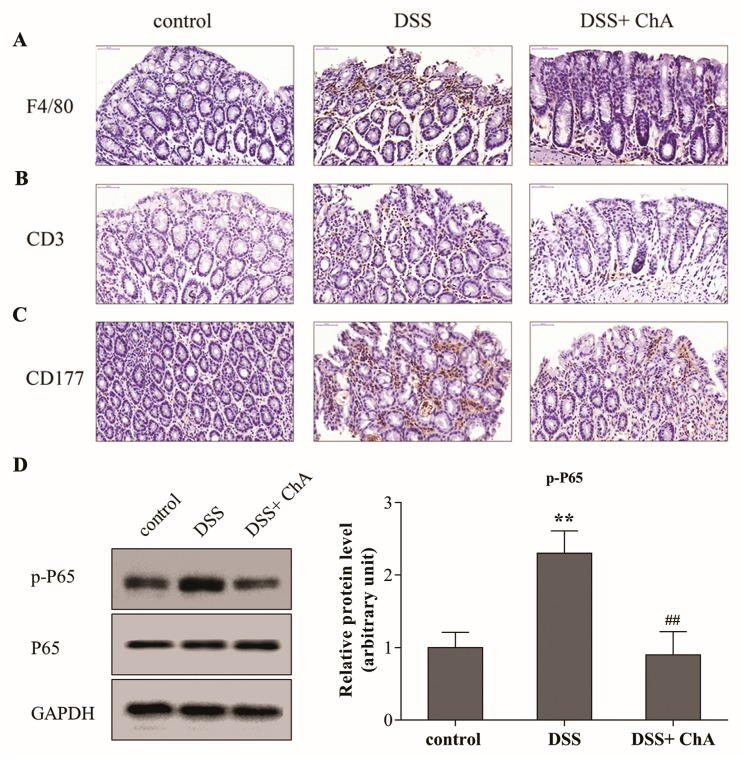
The effects of ChA on colonic infiltration of inflammatory cells and NF-κB signaling in DSS-colitis mice. Representative images of (**A**) F4/80; (**B**) CD3; (**C**) CD177 immunostaining in the distal colon of mice one week after cessation of DSS treatment. Formalin fixed, paraffin-embedded 5 μm cross-sections were stained with respective primary antibody. Scale bar: 50 μm; (**D**) Each protein (80 μg) was determined by immunoblot using specific antibodies. ImageJ software was used to quantify the integrated band intensity, which was transferred to relative values using corresponding GAPDH as an internal reference. The data are expressed as the mean ± SD of three separate experiments from each mouse. ** *p* < 0.01, compared with control group; ^##^ *p* < 0.01, compared with the DSS group.

The expression of F4/80^+^, CD3^+^, and CD177^+^ in distal colonic lamina propria was detected by immunohistochemistry as markers of macrophages, T cells, and neutrophils and infiltration, respectively. Compared to the control mice, DSS triggered an increased infiltration of F4/80^+^ macrophages (Figure 4A), CD3^+^ T cells (Figure 4B) and CD177^+^ neutrophils (Figure 4C) into the colonic lesion area. Treatment with ChA ameliorated the infiltration of T cells, neutrophils and macrophages compared with the group that was treated with DSS alone. Compared with the control group, the expression of p-P65 significantly increased in response to DSS. Meanwhile, ChA could significantly reverse this increase (Figure 4D). These results suggest that NF-κB signaling was activated by DSS, and ChA treatment could inhibit this effect.

### 3.5. The Effects of ChA on Bacterial Diversity in DSS-Colitis Mice

**Figure 5:**
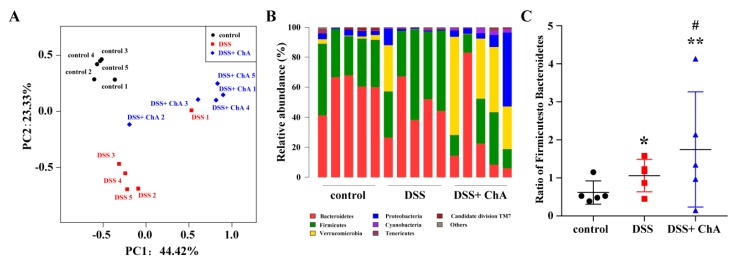
The effects of ChA on microbial composition in DSS-colitis mice. (**A**) Principal coordinate analysis plot of the fecal microbiota from five cages in each group based on the Bray–Curtis; (**B**) Relative abundance of bacterial phyla; (**C**) The ratio of *Firmicutes* and *Bacteroidetes*. * *p* < 0.05, ** *p* < 0.01, compared with control group; ^#^ *p* < 0.05, compared with the DSS group.

The Bray–Curtis dissimilarities were calculated and displayed them by PCoA (Figure 5A)—there was a complete separation of samples by the three treatments. Taxonomic bins at the phylum level showed that the DSS-treated mice treated with DSS showed a trend to harbor higher proportions of *Firmicutes*, and lower proportion of *Bacteroidetes* (Figure 5B). ChA significantly decreased the proportions of *Firmicutes* and *Bacteroidetes* and increased the proportions of *Verrucomicrobia* in DSS-treated mice (Figure 5B,C).

**Figure 6:**
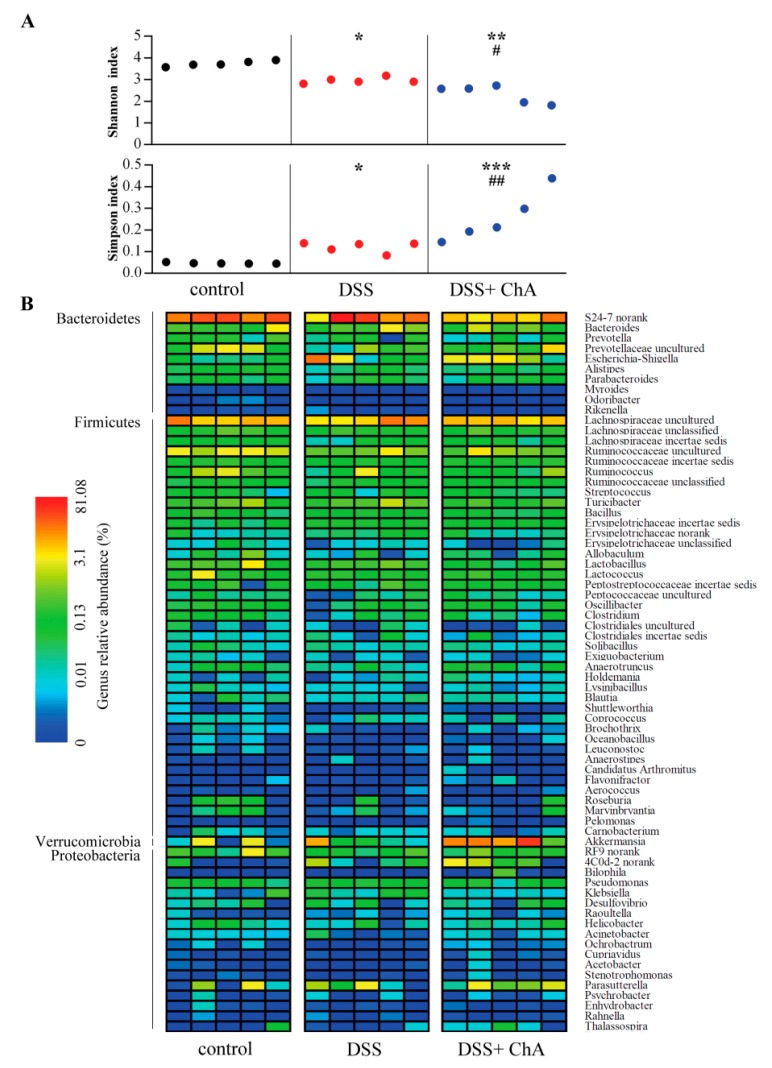
Bacterial α-diversity comparison and heatmap of the relative bacterial species abundance. (**A**) The Shannon and Simpson indices were used to estimate diversity (at a 97% similarity level) of the fecal microbiota in mice; (**B**) Heatmap indicating genus-level changes among controls, DSS, and DSS+ ChA groups. Consistent with alpha diversity indices, clustering analysis of these genera highlighted the apparent differences in their distributions. The relative abundance of each genus is indicated by a gradient of color from blue (low abundance) to red (high abundance). The data represented five samples from respective groups. * *p* < 0.05, ** *p* < 0.01, *** *p* < 0.001, compared with the control group; ^#^ *p* < 0.05, ^##^ *p* < 0.01, compared with the DSS group.

Both Shannon and Simpson indices were calculated to describe within sample diversity. A significant reduction in fecal microbiota diversity was observed in DSS treated mice compared to mice that were fed a standard diet, and ChA treatment significantly enhanced this effect (Figure 6A). In addition, DSS treatment significantly decreased fecal microbiota richness. However, there were no significant differences in the richness indices of DSS and DSS+ ChA group (Figure S2). ChA treatment could increase the abundance of bacterial populations of *Verrucomicrobia* (Figure 6B).

### 3.6. The Effects of ChA on Akkermansia

**Figure 7:**
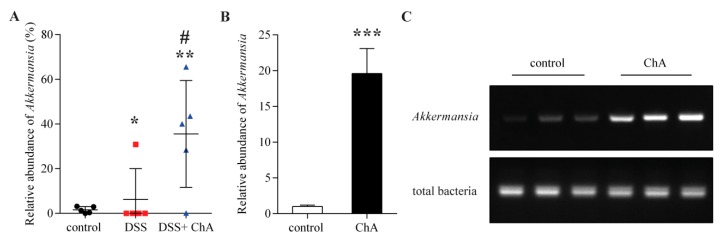
The effects of ChA on *Akkermansia*. (**A**) Comparison of the relative abundance of *Akkermansia* in fecal samples of DSS colitis mice analyzed by 16S rRNA gene sequencing; (**B**) A comparison of the relative abundance of *Akkermansia* in fecal samples of normal mice that were treated with ChA. *Akkermansia* was quantified with qPCR by amplifying fecal DNA with primers specific for *Akkermansia* and universal bacterial primers; (**C**) The polymerase chain reaction products were analyzed by electrophoresis in a 2% agarose gel. * *p* < 0.05, ** *p* < 0.01, *** *p* < 0.001, compared with the control group; ^#^ *p* < 0.05, compared with the DSS group.

ChA treatment significantly increased the relative abundance of *Akkermansia*, which belonged to *Verrucomicrobia* in DSS-treated mice (Figure 7A). To confirm this effect, the relative abundance of *Akkermansia* was detected in normal mice that were treated with ChA. Expectedly, ChA could significantly increase the proportion of *Akkermansia* (Figure 7B,C)

## 4. Discussion

Murine colitis that was induced by DSS in drinking water was one of the well-established experimental models for studying IBD. It has been used to investigate the regulatory mechanisms that reduce inflammation and restore intestinal homeostasis. Preclinical studies that were carried out in the recent past have shown that certain dietary agents, spices, oils, and dietary phytochemicals that are consumed regularly possess beneficial effects in preventing/ameliorating UC [24,25]. The aim of this study was to characterize the effects of ChA on DSS-induced experimental colitis, mainly focusing on the composition of the fecal microbiota and colonic inflammation in mice with DSS-induced colitis.

In this study, 1 mM ChA was ministered to DSS-colitis mice. Considering the stress that could be induced by oral administration, we supplied 10 μL/g (average, 200 μL/mouse) of ChA once a day, yielding ChA doses of 3.54 mg/kg body weight. Habitual coffee consumers generally ingest 500–1000 mg ChA/day (7–14 mg/kg body weight) [12,13]. Previous study had shown that that coffee consumption is a protective factor for UC in Asia-Pacific [14]. We demonstrated a protection against colitis that was induced by DSS in mice with ChA treatment, exhibiting improved weight loss and DAI scores. In addition, the macroscopic and histological evaluation showed that ChA could attenuate the mucosal damage, such as sloughing of epithelial cells and downregulation of mucin. The intestinal mucosa is a physical and metabolic barrier against toxins and pathogens in the lumen. Once the mucosal barrier is breached, the submucosa is exposed to a vast pool of luminal antigens, including food and bacteria, and the innate immune responses are engaged to produce large amounts of cytokines [5,26].

In IBD and experimental colitis, monocytes in blood are recruited to the mucosa and differentiate activated macrophages that produce proinflammatory cytokines, such as TNFα, IL-1, and IL-6 [27,28,29]. To evaluate whether the protection against colitis was associated with the down-regulated production of proinflammatory cytokines, the serum levels IFN-γ, TNF-α, IL-1β, IL-6, and IL-12 were detected. The increased serum levels of IL-6 and IL-17 and the colonic expression of TNF-α and IL-17A were observed in DSS-induced acute colitis [7,30]. ChA inhibited TNF-α and H_2_O_2_-induced IL-8 production in Caco-2 cells. In addition, ChA could suppress the mRNA expression of colonic macrophage inflammatory protein 2 and the DSS-induced IL-1β [15]. In the present study, ChA could significantly decrease IFNγ, TNFα, and IL-6 in the serum of DSS-treated mice. The infiltration of activated neutrophil, macrophages, and T cells is one of the most prominent histological features observed in DSS-induced colitis [5,31]. ChA exhibited anti-inflammatory properties through the reduction of neutrophil infiltration and the inhibition of NF-κB-dependent pathways in mice that were treated with trinitrobenzenesulfonic acid (TNBS), which closely mimics clinical and morphological features of human CD [32,33]. In the present study, treatment with ChA decreased the infiltration of neutrophils, macrophages, and T cells compared with the group that was treated with DSS alone. ChA could also inhibit the activation of NF-κB pathways. Thus, ChA had an anti-inflammatory effect in the colonic mucosa in colitis mice, possibly through suppressing the proinflammatory cytokine secretion.

The accumulating data showed that the composition and diversity of the microbiota are altered in IBD patients or in DSS-treated mice [34,35]. Several studies focused on the clinical improvement of acute DSS-induced colitis by using probiotics and antibiotics in order to modulate the commensal microbiota [36]. Dietary polyphenols could contribute to the maintenance of intestinal health by preserving the gut microbial balance through the stimulation of the growth of beneficial bacteria, and the inhibition of pathogenic bacteria [37]. However, the effect of ChA on fecal microbiota in DSS-induced colitis is still poorly understood. To assess whether the fecal microbiota in DSS-induced coilitis was altered by ChA, the bacterial diversity was characterized by 16S rRNA gene. In the present study, ChA could significantly increase the relative abundance of *Proteobacteria* in DSS colitis. However, the mechanism is still unclear. At the phylum level, a general trend for the reduction of *Bacteroidetes* and increased relative abundance of *Firmicutes* were found in DSS-treated mice versus controls, ChA significantly decreased the proportions of *Firmicutes* and *Bacteroidetes.* These findings suggested that ChA was not exerting a strong antimicrobial effect, however, it could selectively suppress the growth of some members of intestinal microbiota.

*Akkermansia* is a Gram-negative, strict anaerobe and mucin-degrading bacterium that lives in the mucus layer of the intestine and represents 1–3% of the total gut microbiota [38]. *Akkermansia* can regulate the expression of genes that are involved in the host lipid metabolism and epigenetic activation, or the silencing of gene expression, such as fasting-induced adipose factor, Gpr43, histone deacetylases, and peroxisome proliferator-activated receptor gamma [39]. The relative abundance of *Akkermansia* was significantly decreased in IBD patients [40]. Despite the fact that ChA could reduce the microbial diversity, it could significantly increase the proportion of *Akkermansia* in DSS-induced colitis. Our previous study had shown that ChA possess antioxidative activities [41]. The strong oxygen radical scavenging capacity of ChA may provide a survival advantage of *Akkermansia*. Dietary polyphenols promote growth of the gut bacterium, *Akkermansia muciniphila*, and attenuate high fat diet-induced metabolic syndrome [42]. A polyphenol-rich cranberry extract was protected from diet-induced intestinal inflammation that was associated with an increased *Akkermansia* spp. population in the gut microbiota of mice [43]. Previous study has shown that the extracellular vesicles derived from *Akkermansia muciniphila* could protect the progression of DSS-induced colitis [44]. In addition, it could also counteract the high-fat diet-induced decrease of mucus layer thickness [45]. It is possible that a direct trophic effect of ChA on *Akkermansia* precedes the positive effects which are found on the mucus layer integrity.

In summary, the present study demonstrated that dietary ChA could ameliorate DSS-induced acute colitis, resulting in the overall attenuation of macroscopic and histological changes, inflammatory cytokine secretion, and the infiltration of immune cells. Our study further suggested that the ability of the ChA administration to raise the relative proportion of *Akkermansia*, which may be in association with this protective effect, leading us to propose that polyphenols may prevent colitis through a prebiotic effect on the gut microbiota.

## Supplementary Materials

The following are available online at www.mdpi.com/2072-6643/9/7/677/s1, Figure S1: Schematic diagram of the experimental study design, Figure S2: The effects of ChA on the richness of fecal microbiota in DSS-treated mice.

## Abbreviations

ChA	Chlorogenic acid
DAI	Disease activity index
DSS	Dextran sodium sulfate
IBD	Inflammatory bowel disease
OTUs	Operational taxonomic units
PCoA	Principal coordinate analysis
UC	Ulcerative colitis
