# Supplementary material for: Chlorogenic Acid Ameliorates Experimental Colitis by Promoting Growth of Akkermansia in Mice
Source: Nutrients. 2017 Jun 29;9(7):677. doi: 10.3390/nu9070677 (PMC5537792; doi:10.3390/nu9070677)
Supplement: Supplementary file 1 [file nutrients-09-00677-s001.zip › nutrients-195579-Supplementary material.pdf]

Chlorogenic acid ameliorates experimental colitis by promoting growth of *Akkermansia* in mice

Zhan Zhang, Xinyue Wu, Shuyuan Cao, Meghan Cromie, Yonghua Shen, Yiming Feng, Hui Yang, Xiaoping Zou, Lei Li

Supplementary material

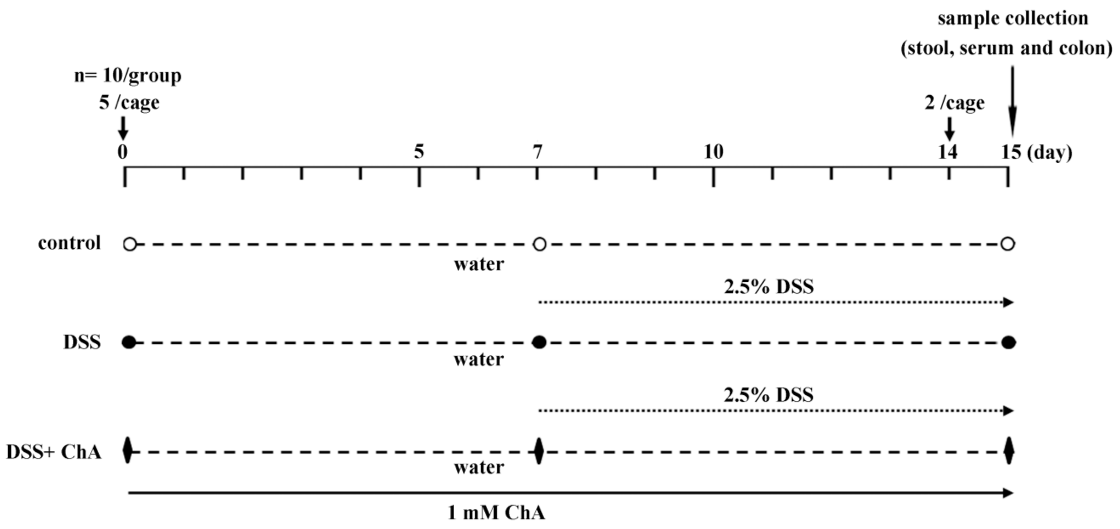

Fig S1. Schematic diagram of the experimental study design. The mice were divided into 3 groups: control, DSS and ChA+ DSS. The control group was given autoclaved water for 15 days; the other 2 groups were given autoclaved water for the first 7 days, and then given water containing 2.5% DSS for the last 8 days. The ChA+ DSS group was orally administered with 1 mM ChA for 15 days.

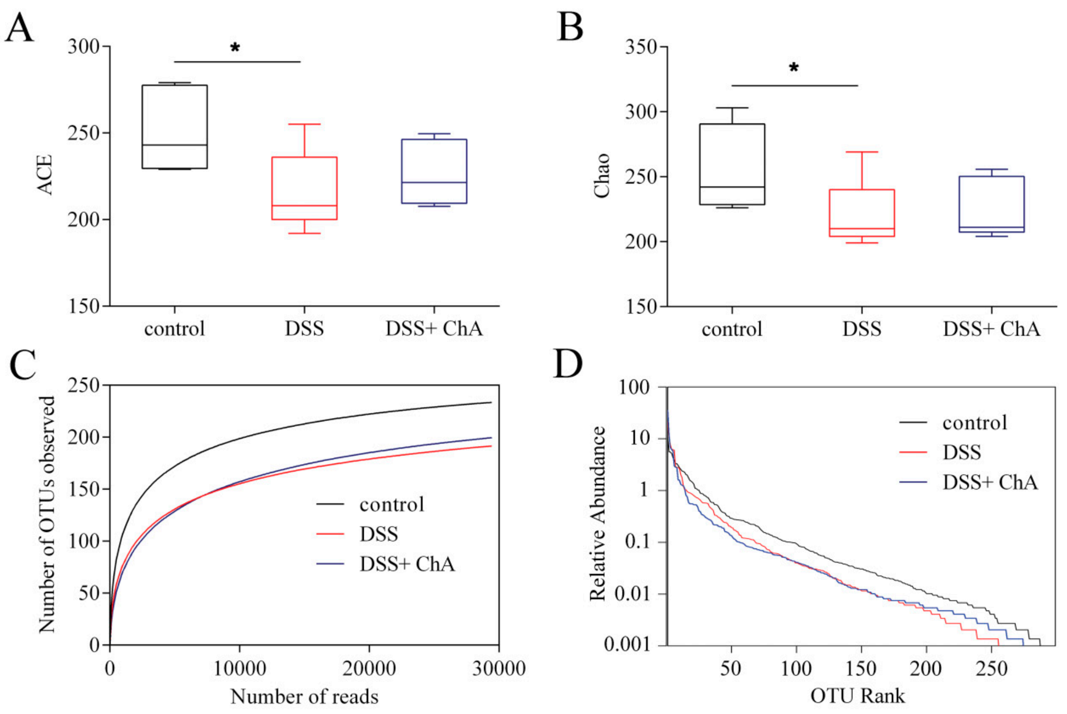

Fig S2. The effects of ChA on the richness of fecal microbiota in DSS-treated mice. (A) ACE and (B) Chao indices were used to estimate richness (at a 97% similarity level) of the fecal microbiota in mice. The richness indices in DSS-treated mice were significantly lower than that in controls, ChA could reverse these effects ($P< 0.01$). (C) Rarefaction curves were used to estimate richness (at a 97% similarity level) of fecal microbiota among the three groups. The vertical axis shows the number of OTUs that would be expected to be found after sampling the number of tags or sequences shown on the horizontal axis. (D) Rank abundance curve of bacterial OTUs derived from the three groups. *$P< 0.05$, compared with control group.
